# Comprehensive Mapping of Common Immunodominant Epitopes in the West Nile Virus Nonstructural Protein 1 Recognized by Avian Antibody Responses

**DOI:** 10.1371/journal.pone.0031434

**Published:** 2012-02-09

**Authors:** Encheng Sun, Jing Zhao, Nihong Liu, Tao Yang, Qingyuan Xu, Yongli Qin, Zhigao Bu, Yinhui Yang, Ross A. Lunt, Linfa Wang, Donglai Wu

**Affiliations:** 1 The Key Laboratory of Veterinary Public Health, State Key Laboratory of Veterinary Biotechnology, Harbin Veterinary Research Institute, Ministry of Agriculture, Chinese Academy of Agricultural Sciences, Harbin, People's Republic of China; 2 Graduate School of Chinese Academy of Agricultural Sciences, Beijing, People's Republic of China; 3 Beijing Institute of Microbiology and Epidemiology, Beijing, People's Republic of China; 4 Australian Animal Health Laboratory, CSIRO Livestock Industries, Geelong, Australia; Washington University, United States of America

## Abstract

West Nile virus (WNV) is a mosquito-borne flavivirus that primarily infects birds but occasionally infects humans and horses. Certain species of birds, including crows, house sparrows, geese, blue jays and ravens, are considered highly susceptible hosts to WNV. The nonstructural protein 1 (NS1) of WNV can elicit protective immune responses, including NS1-reactive antibodies, during infection of animals. The antigenicity of NS1 suggests that NS1-reactive antibodies could provide a basis for serological diagnostic reagents. To further define serological reagents for diagnostic use, the antigenic sites in NS1 that are targeted by host immune responses need to be identified and the potential diagnostic value of individual antigenic sites also needs to be defined. The present study describes comprehensive mapping of common immunodominant linear B-cell epitopes in the WNV NS1 using avian WNV NS1 antisera. We screened antisera from chickens, ducks and geese immunized with purified NS1 for reactivity against 35 partially overlapping peptides covering the entire WNV NS1. This study identified twelve, nine and six peptide epitopes recognized by chicken, duck and goose antibody responses, respectively. Three epitopes (NS1-3, 14 and 24) were recognized by antibodies elicited by immunization in all three avian species tested. We also found that NS1-3 and 24 were WNV-specific epitopes, whereas the NS1-14 epitope was conserved among the Japanese encephalitis virus (JEV) serocomplex viruses based on the reactivity of avian WNV NS1 antisera against polypeptides derived from the NS1 sequences of viruses of the JEV serocomplex. Further analysis showed that the three common polypeptide epitopes were not recognized by antibodies in Avian Influenza Virus (AIV), Newcastle Disease Virus (NDV), Duck Plague Virus (DPV) and Goose Parvovirus (GPV) antisera. The knowledge and reagents generated in this study have potential applications in differential diagnostic approaches and subunit vaccines development for WNV and other viruses of the JEV serocomplex.

## Introduction

West Nile virus (WNV) is a medically important pathogen that is prevalent in many areas around world, including Africa, Europe, Russia, the Middle East, India, Australia and North America [1]. It is serologically classified into the Japanese encephalitis virus (JEV) serocomplex, which includes WNV, JEV, Saint-Louis encephalitis virus (SLEV) and Murray Valley fever virus (MVEV) [2].

WNV is a mosquito-borne flavivirus that primarily infects birds but occasionally infects humans and horses. As such, the virus poses a risk to human health as well as the health of domestic animals and wildlife. In the first half of 2011, WNV was associated with 183 clinical neuroinvasive disease cases and 85 clinical non-neuroinvasive disease cases in humans [3]. The profile of WNV viremia following mosquito-borne infection of birds can vary greatly among different bird species [4]–[6]. Birds that sustained a viremic titer greater than 10^5.0^ plaque forming units (PFU)/ml were considered infectious to *C. pipiens* and *C. quinquefasciatus* [7], [8]. Certain species of birds, including crows, geese, blue jays, ravens, chickens and house sparrows, are considered highly susceptible hosts for WNV, and may be involved in virus transmission through mosquito bites because they develop high levels of viremia after WNV infection [4]–[6], [9]–[21]. Deaths in American crows (AMCR) due to WNV infection were especially prevalent in New York City in 1999 [22], and crow mortality has been adopted as an epidemiological indicator to monitor WNV transmission in the United States and has proven useful in predicting an increased risk for human infection [23]–[25].

Fortunately, commercially raised chickens and turkeys have not been extensively affected by WNV, likely because they are predominantly raised indoors and have limited exposure to mosquito vectors [26]. Although chickens and turkeys can become infected with WNV when experimentally inoculated subcutaneously with WNV, infection results in low viral titers and does not cause clinical disease [27], [28]. Recently, mutations in the prM (prM-I141T) and envelope 40 (E-S156P) genes were shown to mediate the attenuated phenotype of the WNV TM171-03-pp1 virus variant in a chicken macrophage cell line [29]. Once mutated WNV strains were emergent, the strains with high virulence to chickens even causing fatality would also arise, so monitoring epidemiology of WNV infected chickens is necessary. Differences in the course of natural WNV infection in geese as compared to chickens have also been noted, as natural WNV infections could cause severe neurological signs and death in young domestic geese [6], [30]. While the role of domestic geese as a WNV reservoir in an outbreak in Israel is unknown, the infection rates of geese in the Sindbis District of the northern Nile Valley were similar to the rates in buffed-back herons (*Bubulcus ibis ibis*), doves (*Streptopelia senegalensis senegalensis*), and domesticated pigeons (*Columbia livia*) and twice the rate seen in domesticated chickens and ducks (*Anas platyrhynchos*), suggesting that geese may play a role in local WNV ecology [31].

Flavivirus nonstructural protein 1 (NS1) is an important nonstructural protein which plays critical roles in viral RNA replication and the development of flavivirus-associated diseases [32], [33]. Although NS1 protein is not present in the virion of flaviviruses, it can elicit non-neutralizing protective antibodies that inhibit infection through both Fc-γ receptor-dependent and -independent mechanisms [34]. Additional works has shown that passive administration of monoclonal antibody (mAb) against the NS1 protein or active immunization with the NS1 gene or protein confer protection from lethal flavivirus challenge [35]–[39], suggesting that immune responses targeted against the NS1 protein of flaviviruses play an important role in conferring immune protection. A single mAb against flavivirus NS1 protein also could effectively protect against lethal flavivirus challenge [34], [40]–[44]. While anti-E mAb-based therapies could be a promising strategy to control flavivirus infections, sub-neutralizing concentrations of anti-E antibodies have the potential to cause antibody-dependent enhancement (ADE) of flavivirus infections that complicate therapy [45]–[48]. Targeting the NS1 protein using protective antibodies may represent a promising alternative approach.

In future years, it is expected that migratory birds infected WNV will carry the virus to all parts of the United States as well as to Canada, the Caribbean, and Central and South America [49]. Mosquitoes capable of transmitting WNV to susceptible birds exist in each of these regions. The convergence of birds at scarce pools of water also facilitates virus transmission. Diagnostic platforms to monitor WNV infection in domestic avian species, including chickens, ducks and geese, which come into contact with wild waterfowl and birds would be extremely valuable. The rationale exploitation of the antigenic peptide epitopes in the WNV NS1 protein that are targeted by avian antibody responses could provide the basis for such a diagnostic platform.

The aim of our study was to identify immunodominant B-cell epitopes in the WNV NS1 protein that are targeted by the avian immune system using chicken, duck and goose antisera raised against recombinant WNV NS1 protein. The epitopes mapping described in this report will facilitate the development of diagnostic tests for the serological detection of WNV infection and the rationale design of subunit vaccines for WNV and other viruses of the JEV serocomplex.

## Results

### Titers of avian antisera

To generate NS1-reactive polyclonal antisera, chickens, ducks and geese were immunized three times with recombinant WNV NS1 protein. Antisera were collected two weeks after the third immunization for epitopes mapping experiments. Immediately prior to each immunization, serum was collected from each bird to measure the NS1-reactive antibody titers by immunofluorescence assay (IFA). As shown in Table 1, the WNV NS1-reactive antibody titers increased progressively with each sequential immunization in immunized chickens, ducks and geese. As expected, birds that were not immunized with NS1 protein did not have detectable levels of NS1-reactive antibodies at any time point (Table 1). Finally, the titers of the antisera from chicken, duck and goose determined by IFA were 1∶128, 1∶256 and 1∶512, respectively. Therefore, immunization with recombinant WNV NS1 protein elicits high-titer NS1-reactive antibodies in chickens, ducks and geese.

**Table 1:** Determination of the titers of antisera by IFA.

Titer of antisera	Time Points				
	0 week	2nd week	4th week	5th week	6th week
Chicken antisera	<1∶2	1∶16	1∶64	1∶128	1∶128
Chicken control	<1∶2	<1∶2	<1∶2	<1∶2	<1∶2
Duck antisera	<1∶2	1∶8	1∶64	1∶256	1∶256
Duck control	<1∶2	<1∶2	<1∶2	<1∶2	<1∶2
Goose antisera	<1∶2	1∶32	1∶128	1∶256	1∶512
Goose control	<1∶2	<1∶2	<1∶2	<1∶2	<1∶2

### Comprehensive mapping of linear WNV NS1 B-cell epitopes by Western Blot (WB) using avian antisera

We next sought to identify linear epitopes within the WNV NS1 protein that are targeted by the avian antibody responses following immunization with recombinant WNV NS1 protein. We screened a series of 35 partially overlapping peptides covering the entire coding sequence of the WNV NS1 protein using the chicken, duck and goose antisera against purified WNV NS1 protein by WB. The NS1-derived peptides were expressed as MBP-fused polypeptides for screening. As shown in Table 2, the chicken antisera recognized twelve NS1 peptides, the duck antisera recognized nine NS1 peptides, and the goose antisera recognized six NS1 peptides in the series. Three peptides (NS1-3, 14 and 24) were recognized by antibodies in the sera of all three avian species following NS1 immunization; seven peptides (NS1-1, 3, 6, 9, 14, 20 and 24) were recognized by chicken and duck antisera; five peptides (NS1-3, 14, 18, 24 and 27) were recognized by duck and goose antisera; and four peptides (NS1-3, 14, 24 and 26) were recognized by chicken and goose antisera (Table 2). As expected, the control avian sera from unimmunized animals did not detectably react with any of the 35 MBP-fused polypeptides and MBP-tag only (data not shown).

**Table 2:** Identification of linear peptide epitopes in the WNV NS1 protein using antisera from different animals by WB.

Antisera from different animals	Peptides Denomination																	
	*NS1-1*	*NS1-2*	*NS1-3*	*NS1-4*	*NS1-5*	*NS1-6*	*NS1-7*	*NS1-8*	*NS1-9*	*NS1-10*	*NS1-11*	*NS1-12*	*NS1-13*	*NS1-14*	*NS1-15*	*NS1-16*	*NS1-17*	*NS1-18*
**Mouse** *	**+**		**+**	**+**			**+**				**+**			**+**				
**Chicken**	**+**		**+**			**+**		**+**	**+**		**+**		**+**	**+**	**+**			
**Duck**	**+**		**+**			**+**			**+**					**+**				**+**
**Goose**			**+**											**+**				**+**

Antisera from different animals	Peptides Denomination																	
	*NS1-19*	*NS1-20*	*NS1-21*	*NS1-22*	*NS1-23*	*NS1-24*	*NS1-25*	*NS1-26*	*NS1-27*	*NS1-28*	*NS1-29*	*NS1-30*	*NS1-31*	*NS1-32*	*NS1-33*	*NS1-34*	*NS1-35*	*MBP-* *Tag*
**Mouse** *		**+**			**+**	**+**		**+**	**+**				**+**					
**Chicken**		**+**				**+**		**+**										
**Duck**		**+**				**+**			**+**									
**Goose**						**+**		**+**	**+**									

***Note:*** **+** *represents positive reaction with respectively expressed polypeptide using antisera by WB.*

*represents the data cited from EC Sun et al. [57].

### Confirmation of the identified WNV NS1 B-cell epitopes by ELISA using avian antisera

**Figure 1:**
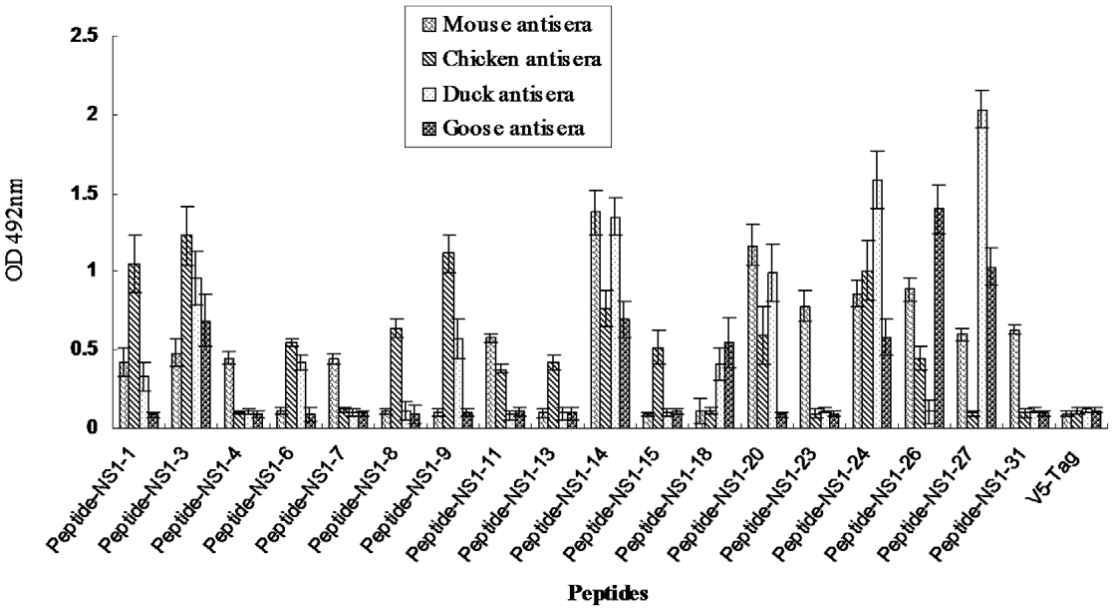
Identification of NS1-derived linear peptide epitopes recognized by murine and avian antibodies generated following immunization with WNV NS1 protein. Murine, chicken, duck and goose sera were collected following immunization with recombinant WNV NS1 protein and were evaluated for reactivity against a series of peptides derived from the WNV NS1 protein by ELISA. Each bar indicates antisera reactivity as determined by the mean absorbance at 492 nm and error bars indicate standard deviation.

To confirm the reactivity of avian antibodies against the peptide epitopes identified by the WB, the candidate polypeptides were synthesized and screened by ELISA using the avian antisera. The chicken, duck and goose antisera displayed the same pattern of reactivity against the WNV NS1-derived polypeptides using an ELISA as was seen in the WB screening against the 35 MBP-fused polypeptides (Figure 1). Importantly, the antisera of all three avian species maintained reactivity against the three common NS1 peptide epitopes by ELISA (Figure 1, Peptide-NS1-3, 14 and 24), suggesting these linear epitopes may be of potential value for the development of serological diagnosis tests. As expected, the control avian sera from unimmunized animals did not detectably react with any polypeptide (data not shown).

### Evaluation of the conservation of three common epitopes among JEV serocomplex viruses

**Figure 2:**
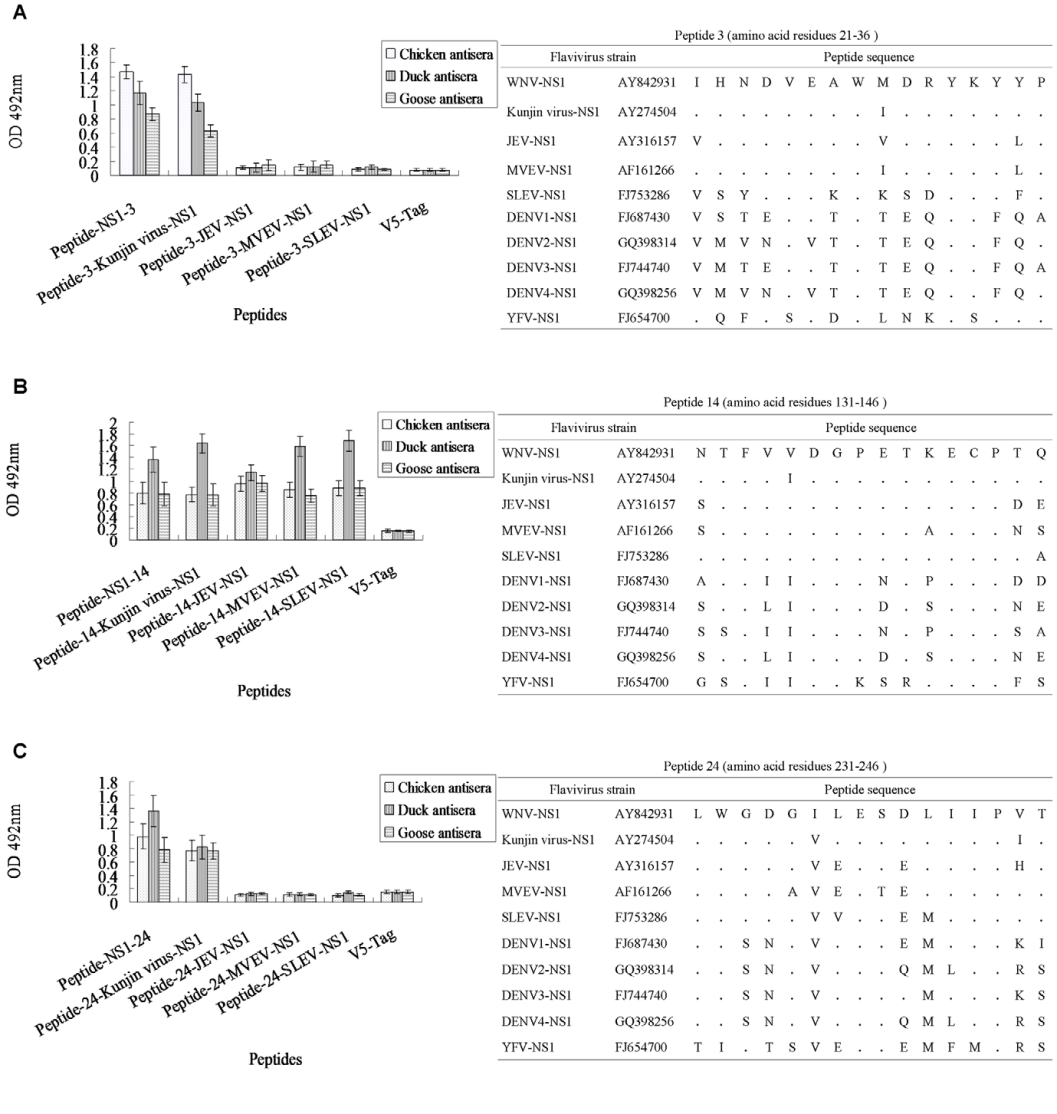
Assessment of the specificity of common avian immunodominant epitopes by ELISA using polypeptides derived from homologous regions of viruses from JEV serocomplex viruses. Chicken, duck and goose antisera immunized with WNV NS1 protein were tested for reactivity against three corresponding polypeptides from other JEV serocomplex viruses by ELISA to identify serotype- and group-specific B cell epitopes. For each polypeptide, the left panel displays the results of ELISA evaluating antibody binding to the JEV serocomplex peptides. Each bar indicates antisera reactivity as determined by the mean absorbance. The right panel depicts the sequence alignments used to identify corresponding polypeptides from representative strains of associated flavivirus isolates. The sequences from DENV1-4 and YFV are shown for comparison.

We next investigated whether the three linear epitopes in the WNV NS1 protein were specific to WNV or conserved among viruses of the JEV serocomplex. To this end, we aligned the NS1 amino acid sequences from viruses of the JEV serocomplex and antigenically related flaviviruses to identify the sequences corresponding to the three commonly recognized linear epitopes identified in the WNV NS1 protein (Figure 2, right panels). The corresponding polypeptides from other viruses of the JEV serocomplex were also synthesized and screened using the avian antisera raised against WNV NS1 protein to evaluate antibody cross-reactivity. The NS1-3 and 24 linear peptide epitopes, located at amino acids 21–36 and 231–246 of the WNV NS1 protein, were specific to WNV, as the three avian antisera did not react with polypeptides derived from the corresponding region of the JEV serocomplex members JEV, MVEV and SLEV (Figure 2A and C, left panels). The NS1-14 epitope, located at amino acids 131–146 of WNV, was common to all the JEV serocomplex viruses tested, as the avian antisera recognized the corresponding polypeptides derived from JEV, MVEV and SLEV (Figure 2B, left panel). As expected, the control avian sera from unimmunized animals did not detectably react with any polypeptide (data not shown).

### Assessment of specificity of the three common peptide epitopes

**Figure 3:**
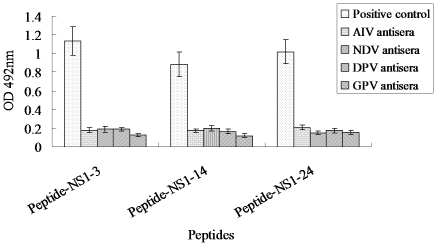
Reactivity of AIV, NDV, DPV and GPV antisera against the common peptide epitopes recognized by avian antisera raised against the WNV NS1 protein. The presence of antibodies reactive against the three common WNV NS1 epitopes in the indicated sera were evaluated by ELISA. Each bar indicates antisera reactivity as determined by the mean absorbance at 492 nm and error bars indicate standard deviation.

Finally, we sought to determine whether antibodies generated against Avian Influenza Virus (AIV), Newcastle Disease Virus (NDV), Duck Plague Virus (DPV) and Goose Parvovirus (GPV) could react with the three common WNV NS1 peptide epitopes that were targeted by avian species following immunization with WNV NS1 protein. AIV, NDV, DPV and GPV antisera did not react with the three WNV NS1 epitopes (NS1-3, 14 and 24), as indicated by an optical density (OD) lower than 0.2 at 492 nm (Figure 3). As expected, the control avian sera did not react with any polypeptide (data not shown). These data indicate that antibodies generated against other viruses, including AIV, NDV, DPV and GPV, do not cross react with the NS1-3, 14 and 24 linear WNV NS1 epitopes, and strongly suggest that these epitopes are specific to WNV and/or JEV serocomplex viruses.

## Discussion

The differential diagnosis of WNV and related flaviviruses has long been a challenge. The practical application of serological diagnostic tests, including the virus neutralization test, the hemagglutination-inhibiting test, ELISA and IFA are limited by biosafety concerns [50]. Antibody cross-reactivity among members of the JEV serocomplex and other flaviviruses can confound the diagnostic outcome [51], [52], especially in geographic regions where several flaviviruses coexist [17]. Several studies have used a WNV NS1-specific mAb to detect natural infections among vaccinated populations and also to differentiate WNV from JEV infections in horses [53]–[56]. These studies illustrate that the NS1 protein can be exploited as a platform for the differential diagnosis of WNV and JEV serocomplex viruses. In addition to the potential utility of antibodies as diagnostic reagents, antibodies that bind epitopes within viral proteins can contribute to viral immunity in several ways, including virus neutralization and opsonization. The identification of antibody binding viral epitopes is required to understand antigenic composition of viral proteins and virus-antibody interactions at a molecular level.

In this study, we immunized three avian species using recombinant WNV NS1 protein and used the antisera to comprehensively map linear B-cell epitopes in the WNV NS1 protein. Following immunization, each avian species developed antibodies against some WNV NS1-derived polypeptides. We identified three WNV NS1-derived polypeptides that were commonly recognized by the antisera of all three avian species tested. We also identified other WNV NS1 peptide epitopes that were differentially recognized by chicken, duck and goose antisera following immunization. Some differences among the antibody specificities of mammalian and avian species were noted when we compared the NS1 epitopes recognized by avian antisera with epitopes previously identified using murine antisera and murine mAbs (Table 2) [57]. Despite some differences in the epitopes recognized among different species, we identified three epitopes that were commonly recognized by both mammalian (murine) and avian (chicken, duck and goose) antibodies. These common WNV NS1 epitopes correspond to WNV NS1 amino acids 21–36, 131–146 and 231–246, respectively, and are referred to as peptides NS1-3, 14 and 24. We confirmed that the NS1-3, 14 and 24 WNV NS1 peptides were commonly recognized by murine and avian antisera in an ELISA format. Further, the high OD at 492 nm that was achieved in the ELISA screening against the three common epitopes with each antiserum strongly suggests that these linear peptide epitopes are immunodominant.

Then we compared the WNV NS1 protein sequences with other viruses of JEV serocomplex and observed some amino acid residues differences between different strains of which some strains reacted with the antisera and others did not. This different reactivity between different polypeptides were associated with the low number of differentiating amino acids of the corresponding epitopes in NS1 protein as shown in Figure 2. The amino acids positions 35 (NS1-3), 237 and 240 (NS1-24) that may be crucial for reactivity of the two WNV-specific epitopes, because the antibodies generated following immunization with the WNV NS1 protein that recognized the NS1-3 and 24 epitopes did not cross react with the corresponding peptide sequences of other JEV serocomplex viruses (Figure 2A and C). More research is needed for further confirmation of these findings. Collectively, these results demonstrate that the two WNV-specific common immunodominant epitopes identified for the WNV NS1 protein can be used in tandem for the rationale design of serological tests to aid the differential diagnosis of WNV and other viruses of JEV serocomplex infection. Additionally, although NS1 protein is absent from the virion, it is expressed on the surface of infected cells and can elicit non-neutralizing antibodies during infection which are effective in protecting infected animals from lethal flavivirus challenge [29], [37]. Therefore, the mapping of antibody epitopes specific to WNV and common to viruses of the JEV serocomplex described in this work may be useful for the future development of avian common subunit vaccines.

Avian species, including crows, blue jays and ravens, are the main reservoir hosts in regions with endemic WNV. As such, these species typically are the initial source of WNV during epizootics occurring outside endemic areas [9], [13], [14], [20], [23]–[25]. Because of their close contact with migratory birds and waterfowl, ducks and geese may play an important role in the epidemiology of WNV. Recent works evaluating the connection between chickens and geese in WNV and infection with other flaviviruses suggested that chickens could serve as an appropriate sentinel animal for monitoring WNV epidemics [5], [6], [28], [30]. Additional, the three common epitopes identified did not react with any antiserum raised against AIV, NDV, DPV and GPV, confirmed the value of using them for serological diagnosis. As it is difficult to obtain sera from avian species that have been naturally infected with WNV or viruses of the JEV serocomplex in China, future works will be needed to confirm that the peptide epitopes identified using polyclonal sera generated against recombinant WNV NS1 protein also serve as antibody epitopes following bona fide infection with WNV.

In summary, our results identified twelve, nine and six peptide epitopes recognized by chicken, duck and goose antibody responses, respectively. Three epitopes (NS1-3, 14 and 24) were recognized by antibodies elicited by immunization in all three avian species tested. We also found that NS1-3 and 24 were WNV-specific epitopes, whereas the NS1-14 epitope was conserved among the JEV serocomplex viruses based on the reactivity of avian WNV NS1 antisera against polypeptides derived from the NS1 sequences of viruses of the JEV serocomplex. The data described in this work will guide the development and clinical application of serological diagnostic tests for WNV and other viruses of JEV serocomplex and also will contribute to avian subunit vaccines development.

## Materials and Methods

### Ethics statement

All animal studies were approved by the Review Board of Harbin Veterinary Research Institute, Chinese Academy of Agricultural Sciences. The Animal Ethics Committee approval number was Heilongjiang-SYXK 2006-032.

### Avian species and proteins

WNV-negative avian species including chickens, ducks and geese were supplied by the Centre of Experimental Animals, Harbin Veterinary Research Institute, Chinese Academy of Agricultural Sciences (CAAS). Purified WNV NS1 protein and 35 partially overlapping, MBP-fused polypeptides covering the entire WNV NS1 protein (NS1-1 to NS1-35) (Data S1) were generated in our laboratory as previously described [57].

### Production and characterization of avian antisera

Chickens, ducks and geese (4 weeks old) were immunized subcutaneously and intramuscularly with purified recombinant NS1 protein in Freund's complete adjuvant (Sigma, St. Louis, MO, USA). The poultry received two boosters with purified NS1 protein in Freund's incomplete adjuvant at 2-week intervals. Animals were bled prior to each immunization, and one and two weeks after the final booster to determine the titer of NS1-reactive antibodies by IFA using WNV antigen slides. Serum collected two weeks after the final booster was used for the epitope mapping experiments described below. The IFA used to determine NS1-reactive antibody titers has been described previously [57]–[59]. Briefly, the primary antibodies were these sera from immunized and unimmunized chickens, ducks and geese. The IFA titers were determined with serial two-fold dilutions from 1∶2 to 1∶1024, and FITC-conjugated goat anti-chicken, rabbit anti-duck or rabbit anti-goose secondary antibodies were added at a 1∶100, 1∶50 and 1∶25 dilution, respectively.

### Comprehensive mapping of epitopes using avian antisera by WB

A series of 35 partially overlapping peptides derived from the amino acid sequence of the WNV NS1 protein were expressed as MBP-fused polypeptides. Antibody reactivity against the MBP-fused polypeptides were screened by WB using chicken, duck and goose antisera. WB was performed essentially as described previously [57]. The primary antibodies from immunized animals or unimmunized animals were added at a 1∶100 dilution, and HRP-conjugated goat anti-chicken, rabbit anti-duck (LICOR Biosciences) or rabbit anti-goose secondary antibodies were added at a 1∶1,000, 1∶500 and 1∶200 dilution, respectively.

### Synthesis of candidate epitopes for confirmatory screening of avian antisera by ELISA

The polypeptides which reacted with avian antisera by WB were synthesized for screening in an ELISA using the avian antisera (Table 3, Shanghai Bootech BioScience&Technology, China). The ELISA was performed as described previously, using synthesized polypeptides as coating antigen (100 ng/well) [57]. The irrelevant polypeptide (V5-Tag: GKPIPNPLLGLDST) and serum from unimmunized animals served as negative controls. All the sera were added at a 1∶100 dilution. HRP-conjugated goat anti-chicken, rabbit anti-duck (LICOR Biosciences) or rabbit anti-goose secondary antibodies were added at a 1∶2,000, 1∶1,000 and 1∶500 dilution, respectively. The cut-off value for the ELISA was determined as the mean OD492 nm values of negative control plus three standard deviations.

**Table 3:** Synthesized polypeptides used to identify linear peptide epitopes recognized by avian antisera.

Peptide Designation	Peptide Sequence
Peptide-NS1-1	DTGCAIDISRQELRCG
Peptide-NS1-3	IHNDVEAWMDRYKYYP
Peptide-NS1-4	RYKYYPETPQGLAKII
Peptide-NS1-6	KEGVCGLRSVSRLEHQ
Peptide-NS1-7	SRLEHQMWEAVKDELN
Peptide-NS1-8	VKDELNTLLKENGVDL
Peptide-NS1-9	ENGVDLSVVVEKQEGM
Peptide-NS1-11	PKRLTATTEKLEIGWK
Peptide-NS1-13	SILFAPELANNTFVVD
Peptide-NS1-14	NTFVVDGPETKECPTQ
Peptide-NS1-15	KECPTQNRAWNSLEVE
Peptide-NS1-18	VRESNTTECDSKIIGT
Peptide-NS1-20	NLAIHSDLSYWIESRL
Peptide-NS1-23	KSCTWPETHTLWGDGI
Peptide-NS1-24	LWGDGILESDLIIPVT
Peptide-NS1-26	RSNHNRRPGYKTQNQG
Peptide-NS1-27	KTQNQGPWDEGRVEID
Peptide-NS1-31	TTESGKLITDWCCRSC
V5-Tag	GKPIPNPLLGLDST

### Analysis of antibody cross-reactivity against the common immunodominant epitopes using polypeptides from the NS1 protein of other JEV serocomplex viruses

Amino acid alignments were performed between the NS1 protein of WNV and other flaviviruses, including the JEV serocomplex members Kunjin virus, JEV, SLEV and MVEV, and the antigenically related flaviviruses DENV1–4 and YFV (Lasergene, DNASTAR Inc., Madison, WI). Based on these amino acid alignments, we identified polypeptide sequences in the JEV serocomplex viruses and related flavivirus that corresponded to the three common immunodominant epitopes identified within the NS1 protein of WNV. Polypeptides of these homologous NS1 regions from the related viruses were synthesized (Table 4, Shanghai Bootech BioScience&Technology, China). The synthesized polypeptides were evaluated for reactivity with avian antisera by ELISA as described above. An irrelevant polypeptide (V5-Tag) and sera from unimmunized avian species served as negative controls. The cut-off value for the ELISA was determined as the mean OD492 nm values of negative control plus three standard deviations.

**Table 4:** Synthesized polypeptides used to assess the cross-reactivity of antibodies that bind common avian immunodominant epitopes in the WNV NS1 protein with corresponding peptides derived from homologous regions of other JEV serocomplex viruses.

Peptide Designation	Peptide Sequence
Peptide-3-Kunjin virus-NS1	IHNDVEAWIDRYKYYP
Peptide-3-JEV-NS1	VHNDVEAWVDRYKYLP
Peptide-3-MVEV-NS1	IHNDVEAWIDRYKYLP
Peptide-3-SLEV-NS1	VSYDVEKWKSDYKYFP
Peptide-14-Kunjin virus-NS1	NTFVIDGPETKECPTQ
Peptide-14-JEV-NS1	STFVVDGPETKECPDE
Peptide-14-MVEV-NS1	STFVVDGPETAECPNS
Peptide-14-SLEV-NS1	NTFVVDGPETKECPTA
Peptide-24-Kunjin virus-NS1	LWGDGVLESDLIIPIT
Peptide-24-JEV-NS1	LWGDGVEESELIIPHT
Peptide-24-MVEV-NS1	LWGDAVEETELIIPVT
Peptide-24-SLEV-NS1	LWGDGVVESEMIIPVT

The polypeptide sequences chosen were the predominant amino acid residues at each position according to the alignment results using all the available strains of certain virus in the NCBI Entrez protein database (http://www.ncbi.nlm.nih.gov/protein/).

### Evaluation of specificity of the three common peptide epitopes

Six AIV, ten NDV, three DPV and three GPV antisera were tested for reactivity against the common epitopes by ELISA. The ELISA was performed as described above, using synthesized polypeptides (Peptide-NS1-3, 14 and 24) as coating antigens. An irrelevant polypeptide (V5-Tag) and sera from unimmunized avian species served as negative controls, the murine WNV NS1 protein positive serum served as positive control [57]. The cut-off value for the ELISA was determined as the mean OD492 nm values of negative control plus three standard deviations.

## Supporting Information

Data S1 **The complementary oligonucleotide pairs encoding 35 overlapping, 16-mer peptides that encompassed the entire NS1 amino acid sequence from the WNV NY99 strain.**
(DOC)
