# Supplementary material for: Comprehensive Mapping of Common Immunodominant Epitopes in the West Nile Virus Nonstructural Protein 1 Recognized by Avian Antibody Responses
Source: PLoS One. 2012 Feb 9;7(2):e31434. doi: 10.1371/journal.pone.0031434 (PMC3276514; doi:10.1371/journal.pone.0031434)
Supplement: Data S1 — The complementary oligonucleotide pairs encoding 35 overlapping, 16-mer peptides that encompassed the entire NS1 amino acid sequence from the WNV NY99 strain. (DOC) [file pone.0031434.s001.doc]

| ***Supplementary Data 1*** |  |  |
| --- | --- | --- |
| **The complementary oligonucleotide pairs encoding 35 overlapping, 16-mer peptides that encompassed the** | | |
| **entire NS1 amino acid sequence from the WNV NY99 strain** | | |
| Designations of | The sequences of oligonucleotides | Coding motifs |
| oligonucleotides |  | (designations) |
| WNV NS1-1-F | 5'-AATTCgacactgggtgtgccatagacatcagccggcaagagctgagatgtggaTAAG-3' | DTGCAIDISRQELRCG (NS1-1) |
| WNV NS1-1-R | 5'-TCGACTTAtccacatctcagctcttgccggctgatgtctatggcacacccagtgtcG-3' |
| WNV NS1-2-F | 5'-AATTCcaagagctgagatgtggaagtggagtgttcatacacaatgatgtggagTAAG-3' | QELRCGSGVFIHNDVE (NS1-2) |
| WNV NS1-2-R | 5'-TCGACTTActccacatcattgtgtatgaacactccacttccacatctcagctcttgG-3' |
| WNV NS1-3-F | 5'-AATTCatacacaatgatgtggaggcttggatggaccggtacaagtattaccctTAAG-3' | IHNDVEAWMDRYKYYP (NS1-3) |
| WNV NS1-3-R | 5'-TCGACTTAagggtaatacttgtaccggtccatccaagcctccacatcattgtgtatG-3' |
| WNV NS1-4-F | 5'-AATTCcggtacaagtattaccctgaaacgccacaaggcctagccaagatcattTAAG-3' | RYKYYPETPQGLAKII (NS1-4) |
| WNV NS1-4-R | 5'-TCGACTTAaatgatcttggctaggccttgtggcgtttcagggtaatacttgtaccgG-3' |
| WNV NS1-5-F | 5'-AATTCggcctagccaagatcattcagaaagctcataaggaaggagtgtgcggtTAAG-3' | GLAKIIQKAHKEGVCG (NS1-5) |
| WNV NS1-5-R | 5'-TCGACTTAaccgcacactccttccttatgagctttctgaatgatcttggctaggccG-3' |
| WNV NS1-6-F | 5'-AATTCaaggaaggagtgtgcggtctacgatcagtttccagactggagcatcaaTAAG-3' | KEGVCGLRSVSRLEHQ (NS1-6) |
| WNV NS1-6-R | 5'-TCGACTTAttgatgctccagtctggaaactgatcgtagaccgcacactccttccttG-3' |
| WNV NS1-7-F | 5'-AATTCtccagactggagcatcaaatgtgggaagcagtgaaggacgagctgaacTAAG-3' | SRLEHQMWEAVKDELN (NS1-7) |
| WNV NS1-7-R | 5'-TCGACTTAgttcagctcgtccttcactgcttcccacatttgatgctccagtctggaG-3' |
| WNV NS1-8-F | 5'-AATTCgtgaaggacgagctgaacactcttttgaaggagaatggtgtggaccttTAAG-3' | VKDELNTLLKENGVDL (NS1-8) |
| WNV NS1-8-R | 5'-TCGACTTAaaggtccacaccattctccttcaaaagagtgttcagctcgtccttcacG-3' |
| WNV NS1-9-F | 5'-AATTCgagaatggtgtggaccttagtgtcgtggttgagaaacaggagggaatgTAAG-3' | ENGVDLSVVVEKQEGM (NS1-9) |
| WNV NS1-9-R | 5'-TCGACTTAcattccctcctgtttctcaaccacgacactaaggtccacaccattctcG-3' |
| WNV NS1-10-F | 5'-AATTCgagaaacaggagggaatgtacaagtcagcacctaaacgcctcaccgccTAAG-3' | EKQEGMYKSAPKRLTA (NS1-10) |
| WNV NS1-10-R | 5'-TCGACTTAggcggtgaggcgtttaggtgctgacttgtacattccctcctgtttctcG-3' |
| WNV NS1-11-F | 5'-AATTCcctaaacgcctcaccgccaccacggaaaaattggaaattggctggaagTAAG-3' | PKRLTATTEKLEIGWK (NS1-11) |
| WNV NS1-11-R | 5'-TCGACTTActtccagccaatttccaatttttccgtggtggcggtgaggcgtttaggG-3' |
| WNV NS1-12-F | 5'-AATTCttggaaattggctggaaggcctggggaaagagtattttatttgcaccaTAAG-3' | LEIGWKAWGKSILFAP (NS1-12) |
| WNV NS1-12-R | 5'-TCGACTTAtggtgcaaataaaatactctttccccaggccttccagccaatttccaaG-3' |
| WNV NS1-13-F | 5'-AATTCagtattttatttgcaccagaactcgccaacaacacctttgtggttgatTAAG-3' | SILFAPELANNTFVVD (NS1-13) |
| WNV NS1-13-R | 5'-TCGACTTAatcaaccacaaaggtgttgttggcgagttctggtgcaaataaaatactG-3' |
| WNV NS1-14-F | 5'-AATTCaacacctttgtggttgatggtccggagaccaaggaatgtccgactcagTAAG-3' | NTFVVDGPETKECPTQ (NS1-14) |
| WNV NS1-14-R | 5'-TCGACTTActgagtcggacattccttggtctccggaccatcaaccacaaaggtgttG-3' |
| WNV NS1-15-F | 5'-AATTCaaggaatgtccgactcagaatcgcgcttggaatagcttagaagtggagTAAG-3' | KECPTQNRAWNSLEVE (NS1-15) |
| WNV NS1-15-R | 5'-TCGACTTActccacttctaagctattccaagcgcgattctgagtcggacattccttG-3' |
| WNV NS1-16-F | 5'-AATTCaatagcttagaagtggaggattttggatttggtctcaccagcactcggTAAG-3' | NSLEVEDFGFGLTSTR (NS1-16) |
| WNV NS1-16-R | 5'-TCGACTTAccgagtgctggtgagaccaaatccaaaatcctccacttctaagctattG-3' |
| WNV NS1-17-F | 5'-AATTCggtctcaccagcactcggatgttcctgaaggtcagagagagcaacacaTAAG-3' | GLTSTRMFLKVRESNT (NS1-17) |
| WNV NS1-17-R | 5'-TCGACTTAtgtgttgctctctctgaccttcaggaacatccgagtgctggtgagaccG-3' |
| WNV NS1-18-F | 5'-AATTCgtcagagagagcaacacaactgaatgtgactcgaagatcattggaacgTAAG-3' | VRESNTTECDSKIIGT (NS1-18) |
| WNV NS1-18-R | 5'-TCGACTTAcgttccaatgatcttcgagtcacattcagttgtgttgctctctctgacG-3' |
| WNV NS1-19-F | 5'-AATTCtcgaagatcattggaacggctgtcaagaacaacttggcgatccacagtTAAG-3' | SKIIGTAVKNNLAIHS (NS1-19) |
| WNV NS1-19-R | 5'-TCGACTTAactgtggatcgccaagttgttcttgacagccgttccaatgatcttcgaG-3' |
| WNV NS1-20-F | 5'-AATTCaacttggcgatccacagtgacctgtcctattggattgaaagcaggctcTAAG-3' | NLAIHSDLSYWIESRL (NS1-20) |
| WNV NS1-20-R | 5'-TCGACTTAgagcctgctttcaatccaataggacaggtcactgtggatcgccaagttG-3' |
| WNV NS1-21-F | 5'-AATTCtggattgaaagcaggctcaatgatacgtggaagcttgaaagggcagttTAAG-3' | WIESRLNDTWKLERAV (NS1-21) |
| WNV NS1-21-R | 5'-TCGACTTAaactgccctttcaagcttccacgtatcattgagcctgctttcaatccaG-3' |
| WNV NS1-22-F | 5'-AATTCaagcttgaaagggcagttctgggtgaagtcaaatcatgtacgtggcctTAAG-3' | KLERAVLGEVKSCTWP (NS1-22) |
| WNV NS1-22-R | 5'-TCGACTTAaggccacgtacatgatttgacttcacccagaactgccctttcaagcttG-3' |
| WNV NS1-23-F | 5'-AATTCaaatcatgtacgtggcctgagacgcataccttgtggggcgatggaatcTAAG-3' | KSCTWPETHTLWGDGI (NS1-23) |
| WNV NS1-23-R | 5'-TCGACTTAgattccatcgccccacaaggtatgcgtctcaggccacgtacatgatttG-3' |
| WNV NS1-24-F | 5'-AATTCttgtggggcgatggaatccttgagagtgacttgataataccagtcacaTAAG-3' | LWGDGILESDLIIPVT (NS1-24) |
| WNV NS1-24-R | 5'-TCGACTTAtgtgactggtattatcaagtcactctcaaggattccatcgccccacaaG-3' |
| WNV NS1-25-F | 5'-AATTCttgataataccagtcacactggcgggaccacgaagcaatcacaatcggTAAG-3' | LIIPVTLAGPRSNHNR (NS1-25) |
| WNV NS1-25-R | 5'-TCGACTTAccgattgtgattgcttcgtggtcccgccagtgtgactggtattatcaaG-3' |
| WNV NS1-26-F | 5'-AATTCcgaagcaatcacaatcggagacctgggtacaagacacaaaaccagggcTAAG-3' | RSNHNRRPGYKTQNQG (NS1-26) |
| WNV NS1-26-R | 5'-TCGACTTAgccctggttttgtgtcttgtacccaggtctccgattgtgattgcttcgG-3' |
| WNV NS1-27-F | 5'-AATTCaagacacaaaaccagggcccatgggacgaaggccgggtagagattgacTAAG-3' | KTQNQGPWDEGRVEID (NS1-27) |
| WNV NS1-27-R | 5'-TCGACTTAgtcaatctctacccggccttcgtcccatgggccctggttttgtgtcttG-3' |
| WNV NS1-28-F | 5'-AATTCggccgggtagagattgacttcgattactgcccaggaactacggtcaccTAAG-3' | GRVEIDFDYCPGTTVT (NS1-28) |
| WNV NS1-28-R | 5'-TCGACTTAggtgaccgtagttcctgggcagtaatcgaagtcaatctctacccggccG-3' |
| WNV NS1-29-F | 5'-AATTCccaggaactacggtcaccctgagtgagagctgcggacaccgtggacctTAAG-3' | PGTTVTLSESCGHRGP (NS1-29) |
| WNV NS1-29-R | 5'-TCGACTTAaggtccacggtgtccgcagctctcactcagggtgaccgtagttcctggG-3' |
| WNV NS1-30-F | 5'-AATTCtgcggacaccgtggacctgccactcgcaccaccacagagagcggaaagTAAG-3' | CGHRGPATRTTTESGK (NS1-30) |
| WNV NS1-30-R | 5'-TCGACTTActttccgctctctgtggtggtgcgagtggcaggtccacggtgtccgcaG-3' |
| WNV NS1-31-F | 5'-AATTCaccacagagagcggaaagttgataacagattggtgctgcaggagctgcTAAG-3' | TTESGKLITDWCCRSC (NS1-31) |
| WNV NS1-31-R | 5'-TCGACTTAgcagctcctgcagcaccaatctgttatcaactttccgctctctgtggtG-3' |
| WNV NS1-32-F | 5'-AATTCtggtgctgcaggagctgcaccttaccaccactgcgctaccaaactgacTAAG-3' | WCCRSCTLPPLRYQTD (NS1-32) |
| WNV NS1-32-R | 5'-TCGACTTAgtcagtttggtagcgcagtggtggtaaggtgcagctcctgcagcaccaG-3' |
| WNV NS1-33-F | 5'-AATTCctgcgctaccaaactgacagcggctgttggtatggtatggagatcagaTAAG-3' | LRYQTDSGCWYGMEIR (NS1-33) |
| WNV NS1-33-R | 5'-TCGACTTAtctgatctccataccataccaacagccgctgtcagtttggtagcgcagG-3' |
| WNV NS1-34-F | 5'-AATTCtatggtatggagatcagaccacagagacatgatgaaaagaccctcgtgTAAG-3' | YGMEIRPQRHDEKTLV (NS1-34) |
| WNV NS1-34-R | 5'-TCGACTTAcacgagggtcttttcatcatgtctctgtggtctgatctccataccataG-3' |
| WNV NS1-35-F | 5'-AATTCgatgaaaagaccctcgtgcagtcacaagtgaatgctTAAG-3' | DEKTLVQSQVNA (NS1-35) |
| WNV NS1-35-R | 5'-TCGACTTAagcattcacttgtgactgcacgagggtcttttcatcG-3' |

***Notes*:** Bases introduced to form the overhanging ends of *EcoR* I and *Sal* I after annealing the two complementary oligonucleotides are shown in capital letters; stop codons TAA are added.
